# Development and Validation of a Machine Learning Model Predicting Arteriovenous Fistula Failure in a Large Network of Dialysis Clinics

**DOI:** 10.3390/ijerph182312355

**Published:** 2021-11-24

**Authors:** Ricardo Peralta, Mario Garbelli, Francesco Bellocchio, Pedro Ponce, Stefano Stuard, Maddalena Lodigiani, João Fazendeiro Matos, Raquel Ribeiro, Milind Nikam, Max Botler, Erik Schumacher, Diego Brancaccio, Luca Neri

**Affiliations:** 1 NephroCare Portugal, Fresenius Medical Care Portugal, 1750-130 Lisboa, Portugal; ricardo.peralta@fmc-ag.com (R.P.); pedro.ponce@fmc-ag.com (P.P.); jfazendeiro.matos@fmc-ag.com (J.F.M.); 2 Clinical & Data Intelligence Systems-Advanced Analytics, Fresenius Medical Care, 26020 Vaiano Cremasco, Italy; mario.garbelli@fmc-ag.com (M.G.); francesco.bellocchio@fmc-ag.com (F.B.); maddalena.lodigiani@fmc-ag.com (M.L.); 3 Global Medical Office-Clinical & Therapeutic Governance Fresenius Medical Care, 61352 Bad Homburg, Germany; Stefano.stuard@fmc-ag.com (S.S.); diego.brancaccio@tiscali.it (D.B.); 4 Nursing Care, Care Operations EMEA, 61352 Bad Homburg, Germany; raquel.ribeiro@fmc-ag.com; 5 Global Medical Office, Global Clinical Affairs, Medical Governance & Digital Health AP, Fresenius Medical Care, Singapore 307684, Singapore; Milind.Nikam@fmc-asia.com; 6 Global Research & Development, Data Solutions, Fresenius Medical Care, 10117 Berlin, Germany; Max.botler@fmc-data-solutions.com (M.B.); erik.schumacher@fmc-data-solutions.com (E.S.)

**Keywords:** machine learning, artificial intelligence, vascular access surveillance, arteriovenous fistula, end stage kidney disease, dialysis, kidney failure

## Abstract

**Background:** Vascular access surveillance of dialysis patients is a challenging task for clinicians. We derived and validated an arteriovenous fistula failure model (AVF-FM) based on machine learning. **Methods:** The AVF-FM is an XG-Boost algorithm aimed at predicting AVF failure within three months among in-centre dialysis patients. The model was trained in the derivation set (70% of initial cohort) by exploiting the information routinely collected in the Nephrocare European Clinical Database (EuCliD^®^). Model performance was tested by concordance statistic and calibration charts in the remaining 30% of records. Features importance was computed using the SHAP method. **Results:** We included 13,369 patients, overall. The Area Under the ROC Curve (AUC-ROC) of AVF-FM was 0.80 (95% CI 0.79–0.81). Model calibration showed excellent representation of observed failure risk. Variables associated with the greatest impact on risk estimates were previous history of AVF complications, followed by access recirculation and other functional parameters including metrics describing temporal pattern of dialysis dose, blood flow, dynamic venous and arterial pressures. **Conclusions:** The AVF-FM achieved good discrimination and calibration properties by combining routinely collected clinical and sensor data that require no additional effort by healthcare staff. Therefore, it can potentially enable risk-based personalization of AVF surveillance strategies.

## 1. Introduction

Arteriovenous fistula (AVF) represents the gold standard vascular access (VA) for haemodialysis (HD). Over time, AVFs may develop dysfunction and lower blood flow due to a series of biological changes that can lead to the formation of a stenosis and subsequent thrombosis. This event has a severe impact on the clinical status of dialysis patients; in the best scenario, endovascular and surgical interventions can restore a satisfactory AVF flow; if not, a central venous catheter (CVC) needs to be placed for interim dialysis access.

Considering the strong negative impact of AVF failure on patient survival, morbidity and quality of life, recent guidelines focused on potential strategies for AVF preservation. The National Kidney Foundation’s (NKF) (KDOQI) Guidelines [1], recommend AVF periodical physical examination (PE), or ultrasound evaluation as primary monitoring methods to detect access dysfunction. However, there is no evidence on the advantages to routine AVF surveillance by measuring intra access blood flow (Qa) [1,2] to improve access patency; nevertheless, its assessment should be considered [3,4].

The controversy concerning the best surveillance strategy to ascertain and evaluate venous stenoses has not yet been solved [5]. The rationale for surveillance is based on the hypothesis that progressive stenosis can be accurately detected by reduced Qa and increased venous pressure (VP) before VA thrombosis occurs [4,6].

Even though both Qa surveillance and ultrasound examination, coupled with pre-emptive correction of hemodynamically significantly reduces the risk of thrombosis and access loss [7,8,9,10,11,12], false positive tests would lead to unnecessary intervention procedures [13] which may ultimately promote further neointimal hyperplasia [14]. No current surveillance method is without pitfalls. Major concerns for Qa surveillance relate to low reproducibility in clinical practice which corresponds to a minimal detectable change as large as 25%, questionable cost-effectiveness as the sole surveillance strategy [15] and suboptimal inter-rater agreement across different measurement techniques [16]. Furthermore, the accuracy in identifying stenosis with Qa varies according to patient characteristics and location [15,17]. On the other hand, ultrasound examination requires significant operator training and skill, may not be readily available in all clinical contexts and may not yield conclusive indications for interventions [18,19]. Structured physical examination has been proposed as a convenient alternative monitoring method. The assessment of PE accuracy in detecting and locating AVF stenosis has shown mixed results; whereas few studies have shown acceptable accuracy in either the diagnosis of outflow and of inflow stenosis [20,21] compared with angiography; few others [22,23] reached opposite conclusions. In addition, a meta-analysis of randomized control trial (RCT) studies showed that blood flow measurement was superior in predicting outcomes [24,25,26]. Furthermore, PE is operator-dependent [27], and has limited long-term prediction power thus explaining why, in a large majority of the cases, many patients may need more frequent surveillance when assuming a rapid AVF deterioration. Taken together, the impact of PE alone on actual prevention of thrombosis is limited [28].

An excellent surveillance method should be quick, easy, accurate, non-invasive, non-operator-dependent and cost-effective. It is clear, that none of the existing methods can fulfil such expectations alone and a one-fits-all approach is not be able to adequately capture the diversity of AVF functional trajectories between and within patients.

In principle, an automatic triage system based on routinely recorded data requiring no additional effort by healthcare professionals may be used to personalize surveillance strategies based on expected risk stratification.

To this end, we sought to develop and validate a risk model based on the machine learning methods predicting the occurrence of AVF failure within three months.

## 2. Materials and Methods

### 2.1. General Description of the Arteriovenous Fistula Failure Model (AVF-FM)

The AVF Failure Model (AVF-FM) aims at predicting the occurrence of a composite AVF failure endpoint (see, Endpoint Definition below) within three months based on routinely recorded clinical information readily available in health information systems for dialysis patients.

The model is based on the XGBoost algorithm, an iterative method where, at each iteration, a new sub-model is added to correct the prediction error of the previous iteration. Each sub-model is an ensemble of decision trees. A decision tree can be roughly described as a flowchart-like structure in which each internal node represents a “discrimination test” on a given attribute (e.g., any clinical parameter or demographic characteristics); each branch of the decision tree represents the result of the discrimination test (i.e., passed or not), and each leaf node represent the probability of the outcome. This probability represents the prevalence of events occurring in each leaf in the training set.

The iterative process ends in accordance with a pre-specified stopping rule (e.g., maximum number of iterations or minimal acceptable average prediction error). The structure of the model is computed as a function optimization process combining the minimization of both training error and model complexity.

We selected XGBoost since it is characterized by a good prediction accuracy in a broad variety of problems coupled with short computational time. Furthermore, SHapley Additive exPlanations (SHAP) analysis [29] enables intuitive model interpretation through an accurate and efficient estimation of the contribution of each input variable to the risk.

### 2.2. AVF-FM Training

The AVF-FM was derived using the information collected in the European Clinical Database (EuCliD^®^, Fresenius Medical Care, Deutschland GmbH, Wendel, Germany), a large, multinational, database including in-centre dialysis patients [30].

**Figure 1:**
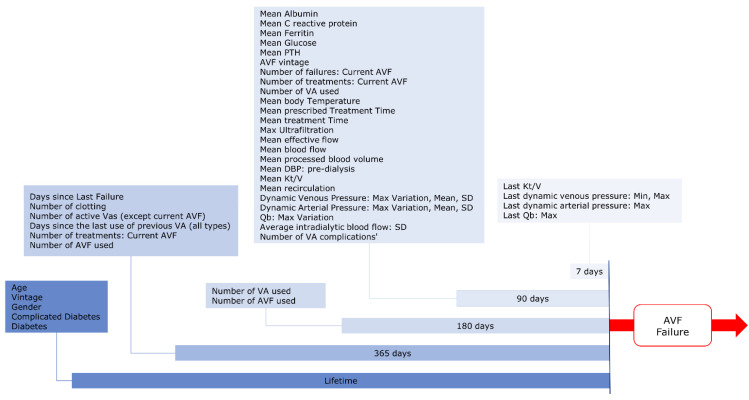
Study Design: the diagram represents the ascertainment period design for different groups of variables.

We enrolled all HD/HDF adult patients in Italy, Spain, and Portugal with at least five treatments performed using AVF as vascular access, in the period January 2015–October 2019 and at least three months of follow-up. Furthermore, we considered only AVFs with more than three months of maturation. The unit of analysis for model development and testing was the patient-quarter. The final dataset included all eligible patient quarters (January, April, July and October) for each year. The ascertainment period for feature computation is represented in Figure 1. To ensure sufficient data completeness, we excluded patients with less than 90 days of ascertainment period before the index date for computation.

### 2.3. Measures

#### 2.3.1. Endpoint Definition

We used a composite endpoint to define AVF failure. EuCliD^®^ has a dedicated module for record AVF failure event. However, reporting in this module may be incomplete. In order reduce the impact of reporting bias, we used a set of proxy variables suggestive of AVF failure. Therefore, we considered as an AVF failure any switch to a different vascular access, the occurrence of procedures aimed at re-establishing AVF patency (e.g., angiography with percutaneous angioplasty, stent placement or surgical AVF revision) and hospitalization due to AVF complications. The exact operative definition of the endpoint variable is described in Supplementary Table S1.

#### 2.3.2. Input Variables

The following classes of variables were considered for model input:

- Socio-demographic and anthropometric parameters;
- Biochemical parameters;
- Vital Signs;
- Dialysis Treatment parameters;
- AVF-related parameters;
- Comorbidities.

We ascertained diabetes by the occurrence of suggestive ICD10 codes according to the Charlson Comorbidity Index (CCI) definition [31]. Additionally, we extracted age, biological sex, dialysis vintage and number of patient’s dialysis access.

#### 2.3.3. Features Generation

We computed several metrics (minimum, maximum, average, standard deviation, slope) for continuous variables (e.g., dynamic venous and arterial needle pressure). Each metric was computed considering different time periods (e.g., last 7, 30, 90 days before index date).

#### 2.3.4. Features Selection

All features have been included in the first model iteration (Supplementary Table S2). Features that provided trivial contribution to model prediction based on feature importance statistics were excluded from the following training iterations. The final model included a total of 46 features derived from 28 variables (Table 1).

#### 2.3.5. Missing Variables Handling

Missing values for the input variables are automatically managed by XGBoost, so no data manipulation was required. The algorithm has proven greater accuracy compared to the standard statistical sample or model based missing data handling methods, as well as other machine learning techniques such as random forest or Bayesian ridge methods. A detailed explanation of how XGboost handles missing variables for a wide range of missingness patterns is beyond the scope of the manuscript and it has been thoroughly described in previous technical publications [32]

### 2.4. Statistical Analysis and Model Performance Evaluation

Model derivation was conducted in a randomly selected partition representing 70% of the original dataset. The final set of variables was obtained as the result of backward stepwise feature selection [33]. Model performance and calibration have been evaluated in the remaining 30% of patients. Model performance was evaluated by concordance statistic and calibration charts. Discrimination was quantified by calculating the area under the receiver operating characteristic curve (ROC AUC) Calibration was visually inspected by plotting observed outcomes incidence by predicted risk score. To evaluate model stability, both training and test has been repeated over 30 random resampling. All statistics are reported as pooled estimates (inverse variance method) and 95% confidence intervals of metrics obtained in the 30 resampling exercises obtained by fixed effect meta-analysis. The importance of input variables for risk prediction was computed using SHAP method. All analysis was performed with Python version 3.7.10, MetaXL^®^ and SAS 9.4^®^.

## 3. Results

### 3.1. Derivation & Test Dataset

The final dataset consisted of 13,369 patients, which provided 113,592 patients-quarters. AVF failure incidence density was 6.6 events/100 patient-quarters or 26.4 events/100 patient years. The AVF failure incidence density in the test set was 6.38 (95% CI: 6.33–6.43). A breakdown of AVF failure events by type is reported in supplementary Table S3. Baseline characteristics of participants are shown in Table 1.

### 3.2. Discrimination and Calibration in the Validation Sample

**Figure 2:**
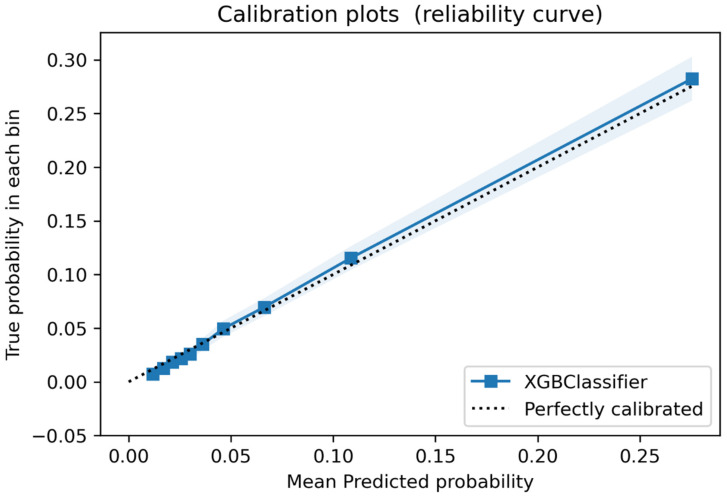
**Calibration Plot.** The calibration plot represents the relationship between predicted probabilities and observed frequency of events in the test dataset. The shaded band represents the 95% confidence interval of the calibration curve. The dotted line represents perfect calibration. The observed calibration curve overlaps with the perfect calibration line over the whole predicted probability distribution.

The final model had a very good discrimination accuracy. The Area Under the ROC Curve (AUC-ROC) for the AVF-FM was 0.80 (95% CI 0.79–0.81). Model calibration showed excellent representation of observed failure risk (Figure 2).

Based on model calibration we established three thresholds identifying 4 risk classes: prevalence and observed event incidence for each risk group is summarized in Table 2.

### 3.3. Feature Analysis

**Figure 3:**
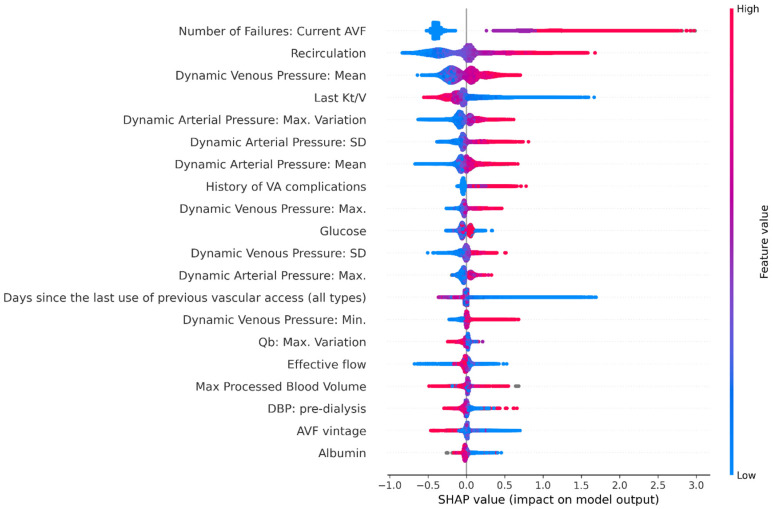
Shapley additive explanations (SHAP) plot showing relative feature importance. Each dot represents one individual subject from the test dataset. Colour Coding: the red colour represents higher value of the variable; the blue colour represents a lower value of the variable. The X axis represent the impact of variables on risk in terms of SHAP values. Positive values suggest direct correlations between risk factors and the occurrence of AVF failures. Negative values suggest inverse correlation between risk factors and the occurrence of AVF failures. Note: AVF, arteriovenous fistula; DBP, diastolic blood pressure; SD, standard deviation; Qb, blood pump flow.

**Figure 4:**
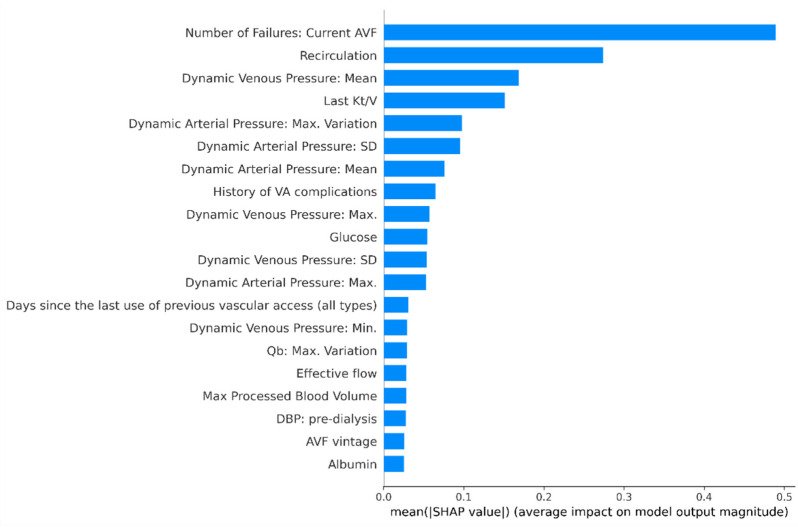
Variable Importance plot. Mean SHAP values represent variable importance plot for the top 20 features in the final model Notes: AVF, arteriovenous fistula; DBP, diastolic blood pressure; SD, standard deviation; Qb, blood pump flow.

The 20 most important data features contributing to performance of AVF failure risk score model, are shown in Figure 3 and Figure 4. Previous history of AVF complications occurred on the vascular access under consideration was the most impactful variable, followed by recirculation and other functional parameters including metrics describing temporal pattern of spKt/V, blood pump flow (Qb), dynamic venous and arterial pressures. Furthermore, AVF vintage, diastolic blood pressure, serum albumin and C-reactive protein were ranked among the top-20 risk contributors.

## 4. Discussion

The wide scale implementation of electronic health record technology has led to an important and unprecedented accumulation of clinical data, and patient information is immediately accessible to computer systems. We exploited the wealth of information stored in the EuCliD^®^ system to derive a machine-learning algorithm for the prediction of AVF failure within three months.

The model showed good discrimination and excellent calibration. To enhance the interpretation and usability of risk estimates yielded by the model we selected three thresholds identifying four distinct risk classes. The largest group was represented by very low risk patients for whom the expected incidence of the composite AVF failure endpoint was remarkably lower than the observed incidence in the whole target population. On the other side of the spectrum there is a small group of patients accounting for less than 1% of the target population with extremely high risk of clinically significant AVF disfunctions within three months. This risk classification can be used to design personalized clinical management workflows. For example, routine monitoring using dialysis parameters and physical examination may suffice for the very low risk group, thus reducing the costs, resource requirements and importantly, unnecessary interventions. Conversely, the very high-risk patient group may be candidate for a more intensive surveillance and clinical review protocol to rule out conditions deserving immediate interventions. In-between, we found two risk classes with moderate and high risk of AVF failure, respectively. For both such classes, the optimal surveillance strategy could be designed to suit the needs and resources of the local clinic, regions, or larger geography. Importantly, accurate risk estimation makes the process of AVF surveillance optimization transparent and reproducible.

Feature analysis disclosed key information to inspect model functioning and enhance score interpretation. Among the 46 input variables, the main contribution to model performance was the past history of failures for the AVF in use, a condition associated with both constitutional proneness to thrombosis and increased AVF vulnerability due to previous surgical interventions aimed at re-establishing patency [34]. In fact, AVF stenosis are one of the most common reasons for repeated endovascular or surgical intervention and are a well-known problem in AV access maintenance. The high re-intervention rate observed (i.e., 2.46 ± 1.40 procedures/patient/year) [35], clearly explains the importance of past history of failure events as a key variable for our model.

One important finding of our study was that the majority of the 15 most important variables in the model were represented by metrics tapping functional parameters of the AVF under examination, namely recirculation rate, dynamic arterial and venous access pressures, effective blood flow and spKt/V. Access recirculation was the second most important contributing feature to risk estimates in our model. The measurement of access recirculation has been used as a non-invasive method based by ultrasound dilution technique (or dilutional-based method) to determine access blood flow (Qa) [36], and stenosis identification. A high degree of access recirculation is one of the factors more importance to identify AVF inflow problems among HD patients and was routinely used for screening of stenosis in 64% from facilities in northern Italy [37]. Access recirculation and poor HD adequacy assessed by spKt/V, may help indicate AV access dysfunction [1]. A recent study by Robert et al. [38] concluded that routine measurements of spKt/V was a quick and straightforward method for early detection of hemodynamically significant AV fistula stenosis.

Similarly, hemodynamic metrics representing the trajectory of dynamic venous and arterial pressures in the dialysis access circuit along time were strong contributors of risk estimates. Alteration of metrics representing the temporal profile of dynamic venous and arterial pressures suggest a high predictive risk of AVF failure. Abnormal dynamic arterial pressure (DAP) may be suggestive of access inflow problems while alterations of dynamic venous pressure (DVP) is associated with outflow stenosis. The incidence of inflow stenosis in patients with AVF from the cases referred to interventional facilities can reach rates of 40% with significant effects in reducing dialysis blood pump flow (Qb) [39]; therefore, combining several AVF dysfunction predictors during the same surveillance evaluation is of paramount importance.

Of note, all such measures are automatically recorded by sensors installed on HD machines and have been used, alone or in conjunction for AVF monitoring [1]. The great advantage of such metrics over routine access flow measurement (Qa) relates to their continuous, effortless availability, since they are measured without any interruption in the patient’s dialysis process, and without time-consuming procedures. Despite Qa has been shown to outperform each of these functional parameters taken alone, this is the first study showing the potential of their combined use for AVF functional assessment. Given that Qa may be consistently available for a minority of patient, we did not include it in the input matrix for model generation. Whether the combination of our risk estimates and Qa provides additional predictive power in selected patients is a matter of further research.

Furthermore, given the strong dependency of risk estimates on AVF functional parameters, our model is sensitive to their changes in AVF and can be used to track risk trajectories over time without any additional data collection burden to the healthcare staff.

Our study has several strengths. The large sample size gathered from multiple dialysis centres across several countries ensured capturing wide diversity in clinical practice and case-mix, two necessary pre-condition for reproducibility and generalizability in machine learning. Additionally, we could leverage on a wide array of clinical variables to characterize patients’ health status including laboratory test results, socio-demographic information, medication, dialysis treatment parameters, comorbidities and data continuously recorded by the dialysis machine during each dialysis session. The evidence regarding risk factors associated with AVF patency loss is still limited. Most studies have small sample size, and a limited set of variables was available [40]. On the contrary, we were able to evaluate the association of AVF patency loss with over 100 clinical parameters and their temporal dynamics, an unprecedented wealth of information. One additional benefit of XGBoost-based algorithm is their inherent explainability, which ensures transparency in clinical decision making. For each patient the model produces SHAP metrics which represent the importance of clinical parameters on risk estimates, allowing independent assessment by the attending physician.

On the other hand, we should acknowledge some limitations as well. Our endpoint definition is a composite outcome including thrombosis, switch to another vascular access, interventions aimed at re-establishing patency in outpatient setting and day hospital admission related to intervention to re-establish patency of the AVF. Despite our operational definition is consistent with the endpoint criteria for AVF patency loss described in the *Recommended standards for reports dealing with arteriovenous hemodialysis accesses* issued by the International Society of Vascular Surgery [41], we rely on data reported by healthcare professionals in clinical practice. Therefore, we cannot rule out the possibility that information bias affected our results. Additionally, our definition reflects medical treatment decision and therefore we cannot exclude that inappropriate surgical intervention have been conducted. This may be reflected in our risk estimates (A detailed description of the endpoint definition is reported Supplementary Table S2). Furthermore, all patients included in our analysis received treatment in the NephroCare network. Despite the multicentre, cross-country design of the study, whether the accuracy and calibration of the AVF-FM can be replicated in centres outside the NephroCare network is a matter of further research.

## 5. Conclusions

The fundamental principle for performing routine vascular access monitoring and surveillance is timely identification and correction of significant stenosis, thus prolonging patency. Current monitoring and surveillance methods remain operator dependent, may be inefficient and may potentially lead to unnecessary interventions.

The AVF Failure Model has shown promising discrimination performance by combining routinely collected clinical as well as sensor data; therefore, the AVF Failure Model can potentially enable risk-based personalization of AVF surveillance strategies. Whether the use of the AVF Failure Model in clinical practice would translate in more efficient care and prolonged access survival is a matter of further clinical testing.

## Figures and Tables

**Table 1:** Patients Characteristics.

Variables	Values
**Socio-Demographics, vital signs and Comorbidities**	
Age (years), median (IQR)	70 (58–78)
Male, n (%)	8971 (67.1)
Body temperature, median (IQR)	36.1 (35.9–36.3)
Renal Replacement Therapy Vintage (months), median (IQR)	17.3 (5.3–59.3)
AVF vintage (months), median (IQR)	9.3 (3.7–42.7)
Diabetes mellitus, n (%)	4959 (37.1)
Complicated Diabetes, n (%)	4238 (31.7)
**Biochemical parameters**	
Albumin (g/dL), mean (IQR)	3.9 (3.6–4.1)
C-reactive protein (mg/L), mean (IQR)	5.1 (2.1–12)
Ferritin (ng/mL), median (IQR)	391 (204–615)
Glucose (mg/dL), median (IQR)	113 (94–152)
PTH (pg/mL), median (IQR)	245 (143–392)
**HD treatment parameters**	
Treatment time (min), median (IQR)	240 (239–242)
Ultrafiltration (L), median (IQR)	3.3 (2.8–4)
Effective blood flow (mL/min), median (IQR)	397 (357–428)
Effective processed blood volume (L), median (IQR)	95.7 (85.1–103.9)
Kt/V, mean (SD)	1.8 (0.4)
Recirculation, median (IQR)	13.9 (11.4–17.7)
**Characteristics of AVF in use**	
Days since the last use of previous vascular access, median (IQR)	74 (38–115)
Number of vascular accesses used in the past 6 months, mean (SD)	1.3 (0.5)
Number of treatments with AVF in the past 6 months, mean (SD)	88.6 (56.3)
**AVF hemodynamic properties**	
Dynamic venous pressure: Mean (mmHg), median (IQR)	182 (165–202)
Dynamic arterial pressure: Mean (mmHg), median (IQR)	−200 (−216–−181)
**AVF failure history and previous adverse events**	
Number of failures: current AVF, mean (SD)	0.6 (1.5)
Days since the last failure, mean (SD)	168 (88.6)
Number of previous thrombosis, mean (SD)	0.4 (1)
Other active vascular access, mean (SD)	0.4 (0.7)
History of vascular access complications, mean (SD)	0.5 (1.4)

All variables were included in the AVF Failure Model. IQR, interquartile range; SD, standard deviation; AVF, arteriovenous fistula.

**Table 2:** Arteriovenous fistula risk score classes.

Risk Class	Prevalence (%)	AVF Failure Risk *	Risk Rate Ratio
Low	45.0 (95% CI: 44.9–45.1)	1.61 (95% CI: 1.57–1.64)	Ref.
Moderate	38.9 (95% CI: 38.8–39.0)	5.29 (95% CI: 5.22–5.36)	3.29 (95% CI: 3.2–3.38)
High	15.7 (95% CI: 15.7–15.8)	21.46 (95% CI: 21.23–21.68)	13.37 (95% CI: 13.04–13.72)
Very high	0.4 (95% CI: 0.3–0.4)	65.76 (95% CI: 63.16–68.45)	41.18 (95% CI: 39.29–43.17)

Risk classes are defined based on three action thresholds of the AVF-FM risk score. Prevalence of each risk class, event rates and risk ratios were estimated in 30 test set obtained as random partition of the original cohort with a 70–30 split. Figures represent pooled estimates (inverse variance method) from 30 random samplings of the of the original cohort. Source figures for each random sampling is reported in Supplementary Table S4. * The AVF Failure Risk is the Positive Predictive Value (events/100 patient-quarters) computed for patients classified in a given risk class; that is PPV = P (Failure|Class). Note: AVF, Arteriovenous fistula.

## Data Availability

The datasets used and/or analysed during the current study are personal health information obtained during provision of healthcare services and cannot be shared to protect their confidentiality in compliance with GDPR regulation.

## Supplementary Materials

The following are available online at https://www.mdpi.com/article/10.3390/ijerph182312355/s1, Supplementary Table S1: Detailed description of the endpoint definition; Supplementary Table S2: All variables included in the first training iteration; Supplementary Table S3: breakdown of AVF Failure causes in our study; Supplementary Table S4: Distribution of AVF-FM risk classes in 30 re-samplings of the test set.
