# Supplementary material for: Development and Validation of a Machine Learning Model Predicting Arteriovenous Fistula Failure in a Large Network of Dialysis Clinics
Source: Int J Environ Res Public Health. 2021 Nov 24;18(23):12355. doi: 10.3390/ijerph182312355 (PMC8656573; doi:10.3390/ijerph182312355)
Supplement: Supplementary file 1 [file ijerph-18-12355-s001.zip › ijerph-1443547-supplementary.pdf]

Supplementary Table S1: All variables included in the first training iteration.

**Initial Model**
Age
Albumin (mean, STD, slope, min, max, last)
Atrial Fibrillation
AVF position
AVF vintage
BCM Overhydration (mean, STD, slope, min, max, last)
BMI
Body Temperature Pre-Dialysis (mean, STD, slope, min, max, last)
Calcium (mean, STD, slope, min, max, last)
Cerebrovascular Disease
Charlson's Index
Chronic Pulmonary Disease
Chronic Rheumatic Heart Disease
Concurrent Temporary catheters
Congestive Heart Failure
Connective Tissue Disorders
Coronary Artery Disease
C-Reactive Protein (mean, STD, slope, min, max, last)
Creatinine (mean, STD, slope, min, max, last)
Dementia
Diabetes
Dialysate Temperature (mean, STD, slope, min, max, last)
Dry Body Weight (mean, STD, slope, min, max, last)
Dyastolic Blood Pressure Post-HD (mean, STD, slope, min, max, last)
Dyastolic Blood Pressure Pre-HD (mean, STD, slope, min, max, last)
Effective Flow (mean, STD, slope, min, max, last)
Etiology: Diabetes
Etiology: Glomerulonephritis
Etiology: Hypertension
Etiology: PKD
Etiology: Tubulo-Interstitial Disease
Ferritin (mean, STD, slope, min, max, last)
Glucose (mean, STD, slope, min, max, last)
Haematocrit (mean, STD, slope, min, max, last)
Haemoglobin (mean, std, slope, min, max, last)
HD Treatment Frequency
HD Treatment Modality
HDL cholesterol (mean, STD, slope, min, max, last)
Heart Rate Post-HD (mean, STD, slope, min, max, last)
Heart Rate Pre-HD (mean, STD, slope, min, max, last)
Height
Hemiplegia
HIV
Hypertension

Intradialytic AVF Temperature Measures (mean, slope, sd, maxmum delta, minimum delta, min, max, last)
Intradialytic Diastolic Blood Pressure (mean, slope, sd, maxmum delta, minimum delta, min, max, last)
Intradialytic Heart Rate (mean, slope, sd, maxmum delta, minimum delta, min, max, last)
Intradialytic Qb (mean, slope, sd, maxmum delta, minimum delta, min, max, last)
Intradialytic Systolic Blood Pressure (mean, slope, sd, maxmum delta, minimum delta, min, max, last)
Intradialytic VA Arterial Pressure Measures (mean, slope, sd, maxmum delta, minimum delta, min, max, last)
Intradialytic VA Venous Pressure Measures (mean, slope, sd, maxmum delta, minimum delta, min, max, last)
Ischemic Heart Disease
Last (Ultra filtrate Volume)/(Dry Body Weight)
Leukocytes (mean, STD, slope, min, max, last)
Liver Disease
Lymphocytes (mean, STD, slope, min, max, last)
Maturation Time
Max (Ultra filtrate Volume)/(Dry Body Weight)
MCH (mean, STD, slope, min, max, last)
MCV (mean, STD, slope, min, max, last)
Metastatic Solid Cancer
Needle Class (mean, mode, Last)
Needle Diameter (mean, mode, Last)
Needle Length (mean, mode, Last)
Needle Tube Length (mean, mode, Last)
Neutrophils (mean, STD, slope, min, max, last)
Non-metastatic cancer
Number of Previous AVFs
Number of Previous VA
OCM-Kt/V (mean, STD, slope, min, max, last)
Other Cardiovascular Comorbidities
Peptic Ulcer
Peripheral Vascular Disease
Phosphate (mean, STD, slope, min, max, last)
Platelet (mean, STD, slope, min, max, last)
Platelet Anti-Aggregants
Potassium (mean, STD, slope, min, max, last)
Previous AVF angiography
Previous AVF complications
Previous AVF interventions
Previous AVF Thromboses
Previous HD adverse events (i.e. intradialytic hypothensions, cramps, etc)
Previous Post-Dialysis adverse events
Proteinuria (mean, STD, slope, min, max, last)
PTH (mean, STD, slope, min, max, last)
Pulmonary Heart Disease
Recirculation (mean, STD, slope, min, max, last)

RRT Vintage
Sex
Smoking
Sodium (mean, STD, slope, min, max, last)
Systolic Blood Pressure Post-HD (mean, STD, slope, min, max, last)
Systolic Blood Pressure Pre-HD (mean, STD, slope, min, max, last)
Time since last therapeutic intervention on the AV
Total cholesterol (mean, STD, slope, min, max, last)
Total Infusion (mean, STD, slope, min, max, last)
Transferrin Saturation (mean, STD, slope, min, max, last)
Treatment Duration (mean, STD, slope, min, max, last)
Treatment Processed Blood Volume (mean, STD, slope, min, max, last)
Treatment Qb (mean, STD, slope, min, max, last)
Ultrafiltrate Volume (mean, STD, slope, min, max, last, CumulatedAUC)
Ultrafiltration Rate (mean, STD, slope, min, max, last)
Ultrafiltration Rate Prescription (mean, STD, slope, min, max, last)
Unspecified Disorder Circulatory System
Urea Reduction Ratio (mean, STD, slope, min, max, last)
Urea Volume (mean, STD, slope, min, max, last)
Weight Post-HD (mean, STD, slope, min, max, last)
Weight Pre-HD (mean, STD, slope, min, max, last)

Supplementary Table S2: Detailed description of the endpoint definition

AVF Failures	AVF failure as defined by the attending physician
	AVF Thrombosis
	Creation of a second VA due to AVF failure as defined by the attending physician
Vascular Access Switch	Switch to another Vascular Access
Interventions aimed at re-establishing patency	Surgical revision of access
	Angiography with PTA
	Angiography with thrombolysis
	Angiography with stent
	Prothesis Interposition
	Patch Angioplasty
	Graft extension
	Graft removal
	Proximal re-anastomosis
	Surgical thrombectomy
	Mechanical thrombolysis (Fogarty)
	Angiography with angioplasty and stent placement
	Percutaneous thrombectomy with angioplasty
	Percutaneous thrombectomy with angioplasty and stent placement
AVF-related Hospitalization	Unspecified complication of internal prosthetic device, implant and graft
	Surgical operation with anastomosis, bypass or graft
	Adjustment and management of vascular access device

Supplementary Table S3: breakdown of AVF Failure causes in our study

AVF Failure as defined by the attending Physician	17.7%
Switch to a second Vascular Access	41.2%
Interventions aimed at re-establishing patency	40.7%
AVF-related Hospitalization	0.4%

Supplementary Table S4: **Distribution of AVF-FM risk classes in 30 re-samplings of the test set.**

		Risk Class			
**Pool Number**	**N**	**Low**	**Moderate**	**High**	**Very High**
Pool1	34529	15340	13738	5330	121
Pool2	34087	14833	13922	5208	124
Pool3	34371	14874	13898	5476	123
Pool4	35079	15736	13669	5559	115
Pool5	34080	15210	13550	5207	113
Pool6	33357	14751	13157	5330	119
Pool7	34032	14947	13461	5491	133
Pool8	34276	15490	13425	5232	129
Pool9	35029	16019	13394	5492	124
Pool10	33027	14598	12834	5494	101
Pool11	33814	15423	12893	5374	124
Pool12	33858	15559	12872	5313	114
Pool13	35482	16446	13346	5550	140
Pool14	34460	16037	12881	5414	128
Pool15	33721	15392	13051	5186	92
Pool16	34474	15689	13342	5312	131
Pool17	34206	15125	13348	5632	101
Pool18	33606	14870	13166	5447	123
Pool19	33953	15032	13519	5283	119
Pool20	33719	15947	12331	5318	123
Pool21	34389	16309	12703	5223	154
Pool22	33775	15362	13060	5237	116
Pool23	33418	15099	12957	5258	104
Pool24	34184	14850	13720	5494	120
Pool25	33411	14687	13408	5193	123
Pool26	34861	15486	13591	5640	144
Pool27	34491	15821	13403	5145	122
Pool28	34493	14991	13899	5489	114
Pool29	34203	15251	13504	5336	112
Pool30	33793	16009	12279	5393	112
**Statistics**					

I^2	94,83
Cochran's Q	560,43
